# Molecular and morphological evidence of the Oriental clade of the tribe Cloresmini (Hemiptera, Coreidae) reveals a new genus: *Gemenesis* gen. nov.

**DOI:** 10.3897/zookeys.1291.189894

**Published:** 2026-09-11

**Authors:** Kun Jiang, Chenguang Zheng, Siqi Wang, Yanfei Li, Juhong Chen, Xiuxiu Zhu, Haoyuan Hu, Wenjun Bu

**Affiliations:** 1 Collaborative Innovation Center of Recovery and Reconstruction of Degraded Ecosystems in Wanjiang Basin CoFounded by Anhui Province and Ministry of Education, School of Ecology and Environment, Anhui Normal University, Wuhu, Anhui 241000, China Collaborative Innovation Center of Recovery and Reconstruction of Degraded Ecosystems in Wanjiang Basin CoFounded by Anhui Province and Ministry of Education, School of Ecology and Environment, Anhui Normal University Wuhu China; 2 College of Life Sciences, Nankai University, Tianjin 300100, China College of Life Sciences, Nankai University Tianjin China; 3 School of Public Health, North China University of Science and Technology, Tangshan 063210, China School of Public Health, North China University of Science and Technology Tangshan China

**Keywords:** 18-28S rDNA, bamboo-feeding insects, Cloresmini, mitochondrion, new combination, new species, phylogeny, taxonomy, ultraconserved elements

## Abstract

Since the establishment of the tribe Cloresmini by Stål in 1873, five genera have been recognized: three distributed in the Oriental region and two in the Oceanian region. In this study, we reconstructed the phylogenetic relationships of 15 species – representing all genera within the Oriental clade – using mitochondrial genomes alongside nuclear 18S and 28S ribosomal DNA loci. Molecular analyses revealed that the genus *Cloresmus* is paraphyletic group (*Notobitus* + (*Cloresmus* + (*Cloresmus* + *Notobitiella*))), a result morphologically supported by the complete fusion of the median fissure and the M vein of the corium. Consequently, we formally establish a new genus *Gemenesis***gen. nov**. and propose two new combinations: *Gemenesis pulchellus***comb. nov**. and *G. yunnanensis***comb. nov**. Furthermore, by integrating molecular and morphological evidence, we describe a new species, *Cloresmus tibiaspinus***sp. nov**., illustrating its diagnostic characters and detailing interspecific genetic distances. This study clarifies the evolutionary relationships and taxonomic boundaries within the Oriental clade of Cloresmini, providing a robust framework for future systematic revisions and evolutionary studies of the entire tribe.

## Introduction

The tribe Cloresmini, commonly referred to as “bamboo-feeding coreid bugs”, was established by Stål in 1873 (initially as the Division ‘Cloresmaria’ Stål 1873), encompassing three recognized genera (Stål 1860, 1873; Costa 1863; Hsiao 1963, 1977). The primary diagnostic characters defined by Stål (1873) for this group included: posterior coxal cavities less deeply excised from the metasternum, with their outer margin strongly diverging from the body’s longitudinal axis and forming an obtuse angle with the posterior margin of the metapleura; a transversely rugose scent gland area; a process extending from the apex of the elevated anterior margin of the scent gland sulcus, not reaching the anterior margin of the metasternum; and a posterior femora thickened and spinose. Subsequently, Hsiao (1963) erected a fourth genus, *Notobitiella* Hsiao, 1963, in China, which is distinguished primarily by its remarkably large parameres in males and the spiny seventh abdominal sternite in females. Later, Brailovsky (2006, 2007) and Brailovsky and Barrera (2007) confirmed a fifth genus based on specimens collected in New Guinea, identifying it as the sister group to the previously described Oceanian genera. To date, the tribe comprises five recognized genera and 43 species: *Cloresmus* Stål, 1860; *Notobitus* Stål, 1860; *Notobitiella*; *Priocnemicoris* Costa, 1863; and *Wasbauerellus* Brailovsky, 2007. Among these, three genera encompassing 11 species are distributed in China.

Phylogenetic studies utilizing ultraconserved elements and mitochondrial genomes suggest that Cloresmini originated approximately 60 million years ago, with a phylogenetic relationship as (Cloresmini + ((Colpurini + Agriopocorini) + (Amorbini + Mictini))) (Forthman et al. 2020; Ye et al. 2022). Preliminary analyses based on the COI gene, indicated that *Notobitiella* and *Cloresmus* share a closer phylogenetic affinity to each other than either does to *Notobitus* (Jiang et al. 2022). Morphologically and biogeographically, Cloresmini is structured into two distinct lineages: an Oriental clade (comprising *Cloresmus*, *Notobitus*, and *Notobitiella*) and an Oceanian clade (comprising *Priocnemicoris* and *Wasbauerellus*), each strictly endemic to its respective realm (Hsiao 1977; Brailovsky and Barrera 2007; CoreoideaSF Team 2026). However, limited taxon sampling and the low phylogenetic informativeness of the COI fragment have hindered deeper evolutionary inference within the tribe. In contrast, molecular phylogenies based on combined mitochondrial genomes and the 18S and 28S ribosomal DNA (rDNA) loci have proven more reliable for delineating species and genera and for resolving phylogenetic relationships (Dong et al. 2022; Zhu et al. 2022; Chen et al. 2024). A robust phylogenetic reconstruction relying on expanded taxon coverage and multi-locus data is urgently needed to overcome these limitations and refine the classification of the tribe.

In this study, we reconstructed the phylogeny of Cloresmini using both mitochondrial genomes and 18S-28S rDNA sequences. Our sampling included 11 ingroup species, representing a comprehensive revision of all genera in the Oriental clade. Phylogenetic analyses prompted a taxonomic reassessment, leading to the establishment of a new genus, *Gemenesis* gen. nov., segregated from the paraphyletic *Cloresmus* based on congruent molecular and morphological evidence. Accordingly, two new combinations are proposed. Furthermore, we provide a refined delineation of the diagnostic characters for all genera within the tribe. Finally, we describe a new species, *Cloresmus tibiaspinus* sp. nov., from Yunnan, China, with detailed illustrations of its key morphological features.

## Material and methods

A total of 33 samples representing 15 species (12 ingroup species of Cloresmini and three outgroup species) were selected for molecular analyses (see Suppl. material 1: table S1). Genomic DNA was extracted from thoracic muscle tissue using the DNeasy Blood & Tissue Kit (QIAGEN, Germany). Whole mitochondrial genomes were sequenced on the Illumina HiSeq 2000 platform (Illumina, USA) with a 350 bp insert library and 150 bp paired-end reads at Novogene (Beijing, China), generating approximately 2 GB of raw data per sample.

Mitochondrial genomes were assembled de novo using MitoZ 2.4 (with default parameters), and to ensure assembly accuracy and reliability, the results were independently verified using IDBA-UD with k-mer values ranging from 40 to 120 bp (Peng et al. 2012; Meng et al. 2019). Transfer RNA (tRNA) genes were annotated using MITOS2 on the Galaxy platform (Donath et al. 2019), applying the invertebrate mitochondrial genetic code. The 13 protein-coding genes (PCGs) were initially identified, and their boundaries were verified and manually corrected using the NCBI ORFfinder. All mitochondrial genomes were assembled and curated in BioEdit, and complete sequences were aligned using MAFFT 7.0 with the L-INS-i algorithm (Katoh 2002). The alignments were manually proofread by comparison with the reference mitogenome of *Cloresmus pulchellus* Hsiao, 1963 (NC_042806), and the two ribosomal RNA (rRNA) genes were confirmed. For phylogenetic analysis, the 13 PCGs were translated into amino acid sequences using MEGA v. 12.0 to ensure accurate reading frames, and the third codon positions were trimmed using PhyloSuite v. 1.2.3 to reduce saturation effects. Pairwise genetic distances based on the COI gene were calculated using the Kimura 2-parameter (K2P) model in MEGA v. 12.0 (Stecher et al. 2025).

To assess phylogenetic signals under different models, we constructed four concatenated mitochondrial datasets from the 33 samples: (1) PCG12: all 13 PCGs with third codon positions removed; (2) PCG123: all 13 PCGs with all codon positions; (3) PCG123RT: all 13 PCGs, two rRNAs, and 22 tRNAs; and (4) PCGAA: the amino acid sequences of all 13 PCGs.

Additionally, nuclear 18S and 28S rDNA sequences were obtained through PCR amplification and Sanger sequencing. These were aligned separately using MAFFT and subsequently combined with the mitochondrial datasets for analysis. The 18S rDNA and the D3 region of 28S rDNA were amplified and sequenced using primers listed in Suppl. material 1: table S2. PCR reactions were performed in 9.5 µl volumes containing 0.5 µl of ddH2O, 7 µl of Es Taq MasterMix (CWBIO, Shanghai, China), 0.5 µL each of the forward and reverse primers, and 1 µL DNA template. Amplification was carried out in a TGRADIENT thermal cycler (Biometra, Germany) under the following conditions: 94 °C for 1 min, followed by 33 cycles of 94 °C for 30 s, 48 °C for 30 s, and 72 °C for 30 s, with a final hold at 4 °C to preserve the DNA (Li et al. 2012). Target products were purified using the Agarose Gel DNA Purification Kit (TAKARA, Dalian) and bidirectionally sequenced with the original PCR primers by BGI Company. These sequences were aligned separately using MAFFT and concatenated into an 18S-28S matrix, which was subsequently combined with the mitochondrial datasets to verify the reliability of the mitogenomic tree topologies.

Phylogenetic analyses were performed using maximum likelihood (ML), and Bayesian inference (BI) methods based on two sources of molecular data: the mitochondrial genomes and the nuclear 18S-28S rDNA. ModelFinder was used to determine the best-fit model (Kalyaanamoorthy et al. 2017). The ML tree was reconstructed with 1000 bootstrap replicates in IQ‐TREE v. 2 (Minh et al. 2020), based on the optimal partitioning model. The BI tree was constructed using four independent Markov Chain Monte Carlo (MCMC) searches in MrBayes v. 3.2.7a (Ronquist et al. 2012), with each search run for 2,000,000 generations, with sampling performed every 1000 generations until the average standard deviation of split frequencies fell below 0.01, discarding the first 25% of the trees as burn-in. Phylogenetic trees were visualized and annotated in iTOL (https://itol.embl.de/).

Guided by the phylogenetic results, we carefully examined the morphological characters of over 800 alcohol-preserved and dried specimens. Subsequently, we conducted a comprehensive revision of Cloresmini specimens in the Institute of Entomology, College of Life Sciences, Nankai University, Tianjin, China (NKU). Photographs were taken with a Canon EOS 5D Mark II camera and focus-stacked with Helicon Focus software. All specimens were deposited in NKU. Morphological terminology follows Hsiao (1963) and Brailovsky and Barrera (2007).

## Resuts

### Molecular analysis

**Figure 1.**
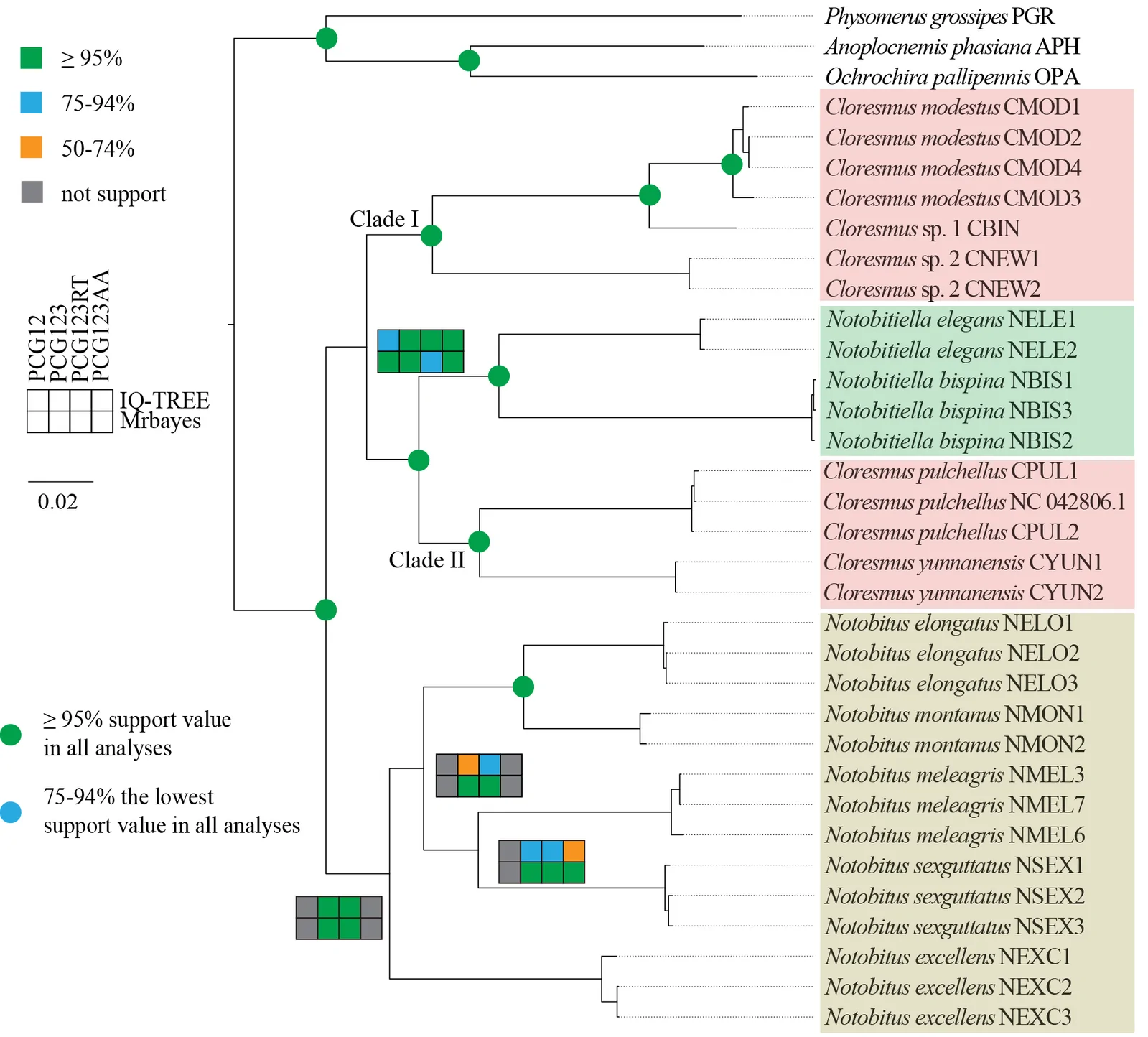
Phylogenetic tree of Cloresmini constructed using mitochondrial genome data (13 protein-coding genes + two rRNAs + 22 tRNAs, PCG123RT). Species marked with the same colour belong to the same genus.

For mitochondrial data: Compared with genus *Notobitus*, the support value of the monophyletic group composed of *Cloresmus* and *Notobitiella* was relatively robust. These datasets consistently demonstrated that *Cloresmus* is a paraphyletic group: (*Cloresmus* Clade I + ((*Notobitiella elegans* Hisao, 1963 + *Notobitiella bispina*) + *Cloresmus* Clade II)) (Fig. 1). Within the genus *Notobitus*, the topology showed inconsistencies across different datasets. Specifically, the phylogenetic positions of *N. excellens* Distant, 1879, *N. meleagris* Fabricius, 1787, and *N. sexguttatus* Westwood, 1842 remained unresolved (Suppl. material 1: figs S1–S3). In contrast, *N. montanus* Hsiao, 1963 and *N. elongatus* Hsiao, 1977 were always recovered as a sister group.

**Figure 2.**
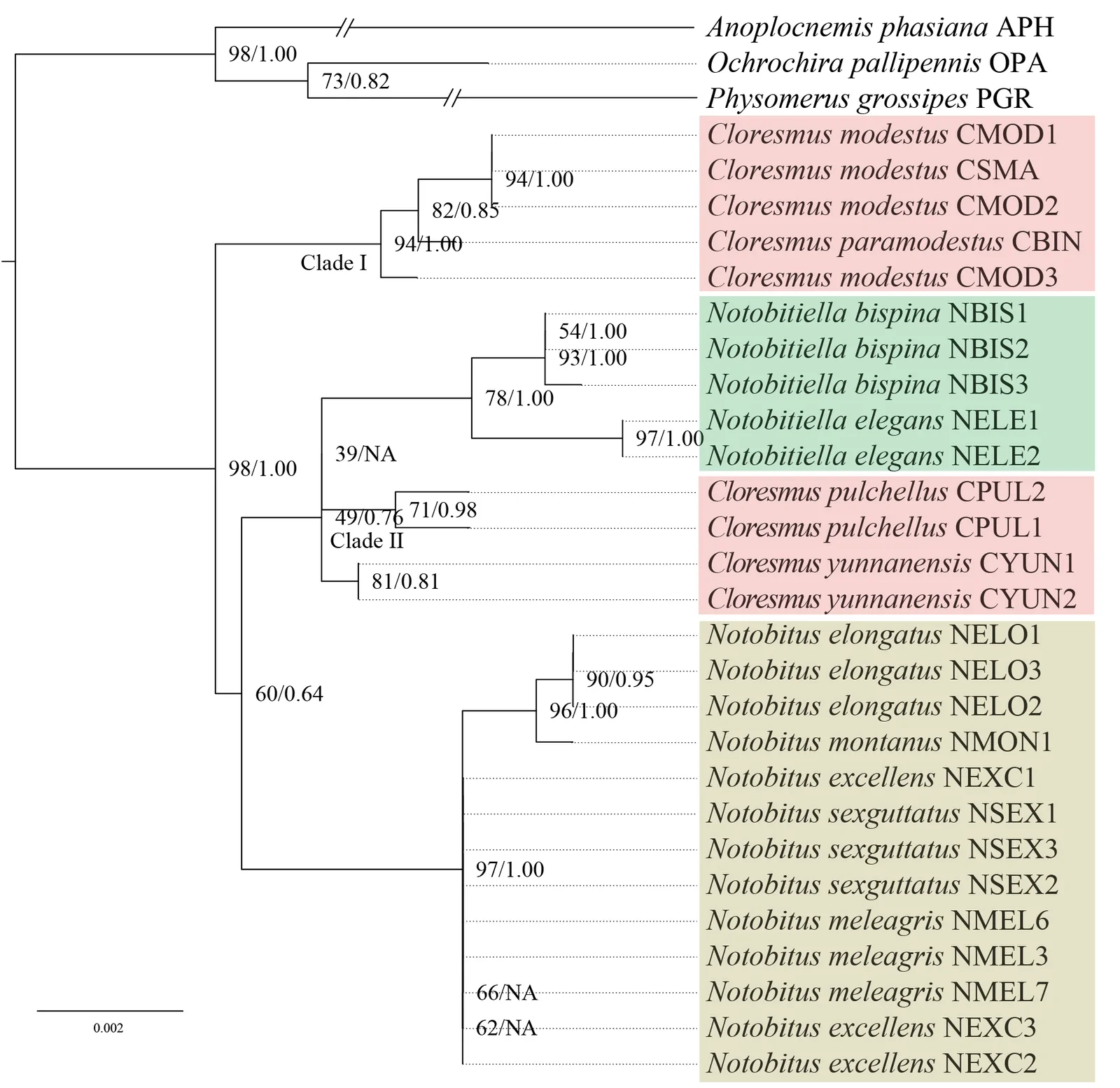
Phylogenetic tree of Cloresmini constructed using 18S-28S rDNA data. Species marked with the same colour belong to the same genus. Numbers on the nodes are bootstrap values for IQ‐TREE and Bayesian posterior values for MrBayes.

For 18-28S rDNA data: Amplification of 18-28S rDNAs failed for two dried specimens: *Cloresmus* sp. 2 and *N. montanus* NMON2. The support value at the tree nodes indicated that these sequences lacked sufficient phylogenetic signal to fully resolve the relationships among species (Fig. 2). However, the results strongly supported that *Cloresmus* was a paraphyletic group (ML = 98; BI = 1.0), and *Cloresmus* Clade II and *Notobitiella* having a closer relationship. The molecular data robustly supported the monophyletic group (*C. yunnanensis* Hsiao, 1963 + *C. pulchellus* Hsiao, 1963) as sister group to the genus *Notobitiella* rather than other *Cloresmus* species.

Analysis of COI genetic distances revealed that interspecific divergence within the tribe Cloresmini ranged from 8.2% to 16.9%. Most pairwise comparisons exceeded 10%, with the exception being the distance between *N. elongatus* and *N. montanus* (8.2%). The COI genetic distance between *Cloresmus* sp. 1 and other species in the genus *Cloresmus* ranged from 5.7% to 6.3%. For *Cloresmus* sp. 2, this divergence ranged from 14.4% to 14.9% (Suppl. material 1: table S3). These genetic divergence levels suggest that *Cloresmus* sp. 1 and sp. 2 may represent distinct new species (Zhang et al. 2017; Zhang and Bu 2022). However, in the present study, we conservatively treat *Cloresmus* sp. 1 as a morphological variant of *Cloresmus modestus* Distant, 1901; formal taxonomic recognition of this taxon requires additional sampling.

### Morphological revision

Our morphological revision revealed distinct diagnostic differences in the corium veins between the two clades of *Cloresmus*. The median fissure and the M vein of the forewing are posteriorly fused but anteriorly separated in *Cloresmus* Clade II, whereas they are completely fused in *Cloresmus* Clade I. The type species of the genus *Cloresmus*, *C. signoreti* Stål, 1860, exhibits the same vein pattern as Clade I (Suppl. material 1: fig. S1). Consequently, *Cloresmus* Clade II is herein erected as a new genus.

Further morphological examination of *Cloresmus* sp. 2 revealed distinct morphological differences from *C. modestus*, indicating that it represents a new species. *Cloresmus* sp. 1 has a pair of unequally sized spines (one large and one small) arranged side by side on the tibia, which is the only diagnostic character distinguishing it from *C. modestus*. However, owing to the availability of only a single specimen, it is difficult to determine the stability of this trait within a population. Therefore, we conservatively refrain from describing it as a new species at present.

### Taxonomy

#### Cloresmini

Taxon classificationAnimaliaHemipteraCoreidae

Stål, 1873

4EF3FE17-F740-501D-A5E4-CDEF32525A69

##### Type genus.

*Cloresmus* Stål, 1860.

##### Revised diagnosis.

Following the present study, the tribe Cloresmini comprises six genera: *Cloresmus* Stål, 1860; *Notobitus* Stål, 1860; *Notobitiella* Hsiao, 1963; *Priocnemicoris* Costa, 1863; *Wasbauerellus* Brailovsky, 2007, and *Gemenesis* gen. nov. Members of this tribe are medium to large-sized, relatively narrow, and parallel-sided, with some species exhibiting a metallic lustre. The pronotum is horizontal or nearly horizontal, with rounded lateral angles. The male femora are relatively thick but do not gradually enlarge toward the apex, and they usually bear long spines on the ventral surface. *Gemenesis* gen. nov. can be distinguished from *Cloresmus* by the median fissure and M vein being fused apically but separated anteriorly, whereas in *Cloresmus*, they are completely fused. *Gemenesis* can also be distinguished from *Cloresmus* by the basoventral surface of the hind femora being armed with a row of small spines, whereas the latter lacks distinct spines.

### Key to the known genera of Cloresmini (Brailovsky & Barrera, 2007)

**Table:** 

1	Fore femur ventrally unarmed or with tiny subapical spine; abdominal sternite VII of female complete, without plica and fisura (female of *Wasbauerellus* unknown); middle third of pronotum with low longitudinal groove, running behind calli to posterior margin; mesosternum with median longitudinal groove at the anterior third; buccula situated after insertion of antennae, and not produced anteriorly	**2**
–	Fore femur with two rows of ventral spines; abdominal sternite VII of female with plica and fissura; middle third of pronotum lacking a median longitudinal groove, mesosternum with or without median longitudinal groove in anterior third; buccula situated before the insertion of antennae, and produced anteriorly	**3**
2	Posttylar depression deep, forming a single sulcus, anterior angles blunt, not exposed; humeral angles obtuse or weakly expanded; hind femora with large acute spine near midline, posterior border of metapleura not raised above the surface, hind tibiae curved at very basal third, and the inner face not expanded at midline, and armed with short and stout spines	***Priocnemicoris* Costa, 1863**
–	Posttylar depression inconspicuous; anterior angles produced forward as rounded expansion; humeral angles expanded, subangulate; hind femora lacking a large and acute spine near midline; posterior border of metapleura raised above the surface, hind tibiae curved, the inner face at the midline slightly expanded, and armed with large and stout spine	***Wasbauerellus* Brailovsky, 2007**
3	Rostral segment I longer than segment II, exceeding the posterior margin of the head	***Notobitus* Stål, 1860**
–	Rostral segment I not longer than segment II, not exceeding the posterior margin of the head	**4**
4	Lateral margin of pronotum with verrucous protuberance; median fissure and M vein of forewings corium completely fused	***Cloresmus* Stål, 1860**
–	Lateral margin of pronotum rounded and smooth, without verrucous protuberance; median fissure and M vein posteriorly fused but anteriorly separated	**5**
5	Body cylindrical, with rostral segment II reaching the middle of the prosternum	***Notobitiella* Hsiao, 1963**
–	Body flat, with rostral segment II reaching the base of head	***Gemenesis* gen. nov**.

#### Gemenesis

Taxon classificationAnimaliaHemipteraCoreidae

Jiang & Bu gen. nov.

5BAB5B6F-48C2-5B45-B6FD-FA858BE8FF1E

https://zoobank.org/F5ED8081-5992-4A87-9551-95A69C6773C9

##### Chinese common name.

金绿竹缘蝽属.

##### Gender.

Masculine.

##### Type species.

*Gemenesis yunnanensis* comb. nov. (*Cloresmus yunnanensis* Hsiao, 1963).

##### Etymology.

Species of this genus are found on bamboo shoot apices, shining with a gem-like lustre. Therefore, the generic name is derived from the Latin word for “gem”, reflecting this characteristic.

##### Remarks.

Prior to this study, *Cloresmus* and *Gemenesis* gen. nov. were treated as a single genus based on shared morphological characters of the rostrum, hind coxa, and the male genital capsule. Guided by our molecular phylogenetic results, a subsequent morphological re-examination demonstrated that they can be reliably distinguished by the following characters. In *Cloresmus* species, the median fissure and M vein of the corium of the forewing are completely fused, and the lateral margin of the pronotum has verrucous protuberances. However, in *Gemenesis* species, the median fissure and M vein are fused posteriorly but separated anteriorly, and the lateral margin of the pronotum is rounded and smooth. These characters are also exhibited by *Notobitiella*, which is the sister group of *Gemenesis*. In addition, body colour can also be used to distinguish between *Cloresmus* and *Gemenesis* to some extent. Species of the former genus, which are typically found in the basal sheaths of mature bamboo, are frequently black or brown. By contrast, members of the latter genus occur on bamboo shoot apices and usually have a green, metallic body. It should be noted that this ecological and coloration pattern is currently based solely on Chinese specimens and requires further validation through broader geographical sampling.

##### Revised diagnosis.

***Size and colour***: Body small to medium-sized, exhibiting metallic green or blue lustre, densely covered with punctures and short fine hairs.

***Head***: Metallic, width approximately equal to length, covered with sparse short hairs; tylus exceeding juga; eyes large and protruding; ocelli red and protruding; vertex roughly level with eyes, without a posttylar depression; antennae cylindrical; antenniferous tubercles protruding; first three segments covered with black bristles, segment I slightly thickened, segment IV longest, evenly curved, light yellow with apical half darkened and densely covered with short fine hairs; buccula produced anteriorly, situated anterior to the antenniferous tubercles; rostrum covered with short fine hairs, reaching only the anterior margin of the mesosternum; rostral segment II reaching the base of the head.

***Thorax***: Pronotum flat, densely covered with punctures and short hairs; collar concolorous with the rest of the pronotum; calli smooth with a shallow pit on the midline; lateral margins of the pronotum rounded and smooth, without verrucous protuberances; posterior margin of pronotum slightly depressed; scutellum rugose, approximately equilateral triangular; corium densely covered with punctures and short hairs; median fissure and the M vein of the corium posteriorly fused, but anteriorly separated; inner margin of hind femora armed with rows of small spines, with males usually bearing a prominent long spine in the middle; hind femora somewhat flattened in some specimens; inner margin of hind tibiae toothed, dorsally grooved; scent gland orifice protruding.

**Figure 3.**
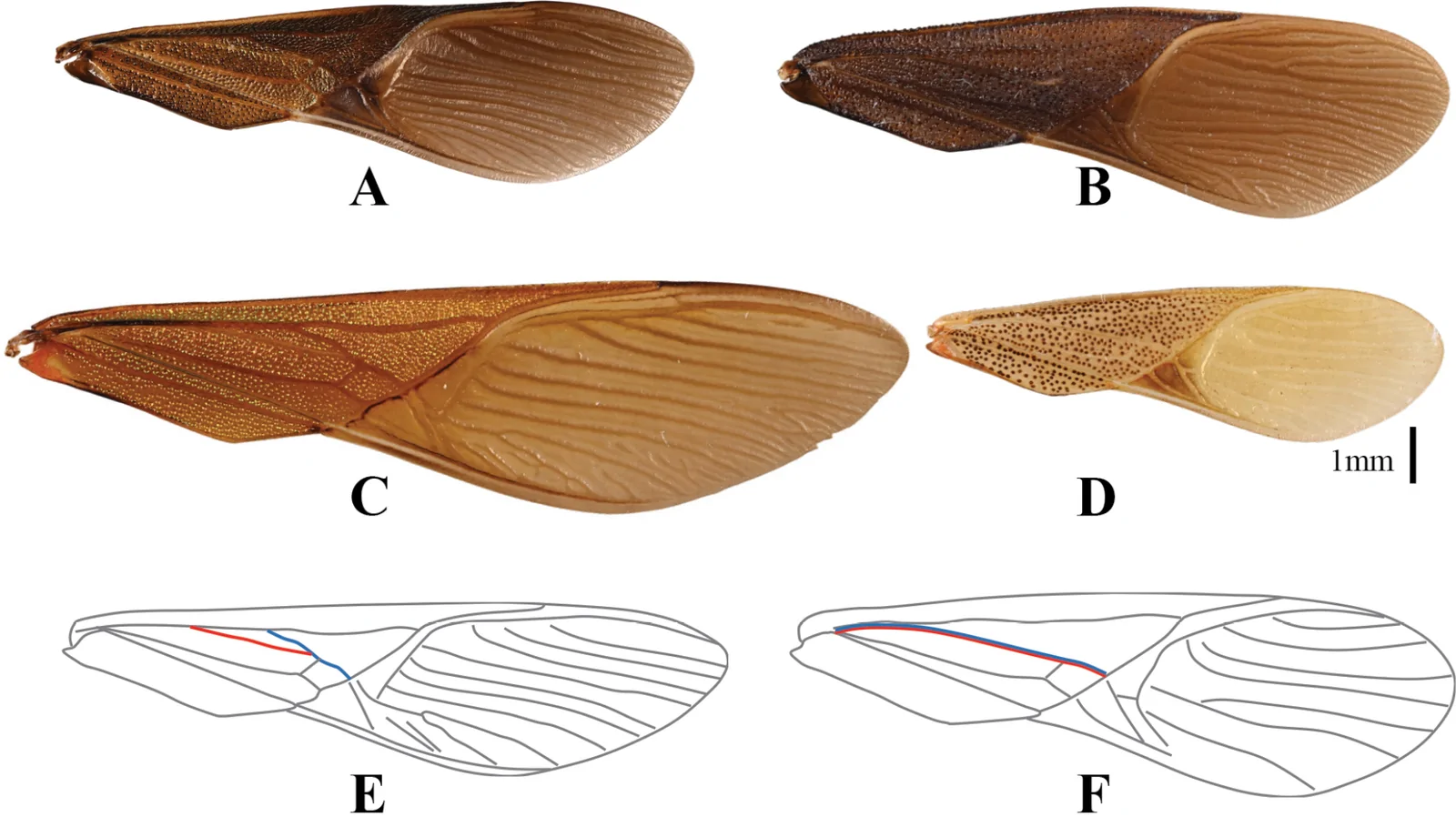
The hindwing venation of the genus *Gemenesis* and *Cloresmus*. **A**. *G. yunnanensis* comb. nov.; **B**. *C. modestus*; **C**. *G. pulchellus* comb. nov.; **D**. *C. tibiaspinus* sp. nov.; **E, F**. Represent the genus *Gemenesis* and *Cloresmus*, respectively. The red line indicates the median fissure, and the blue line indicates the M vein.

***Abdomen***: Abdomen exceeding forewing width, with connexiva visible dorsally; posteroventral margin of the male genital capsule concave and covered in bristles; female sternite VII sheet-like, with plica and a fissura.

##### Diversity and distribution.

China (Yunnan, Guangdong and Guangxi), Vietnam, Thailand and Myanmar.

###### Key to species of *Gemenesis* gen. nov.

**Table:** 

1	Body relatively large (17.0–21.4 mm); dorsum bright metallic green; antennal segment IV yellow on basal half, black on apical half; venter reddish brown; connexivum unicolorous	***G. pulchellus* comb. nov**.
–	Body relatively small (14.4–18.3 mm); dorsum dull metallic green; antennal segment IV entirely yellow; venter orange-yellow; connexivum with basal half pale and apical half black	***G. yunnanensis* comb. nov**.

#### Cloresmus

Taxon classificationAnimaliaHemipteraCoreidae

Stål, 1860

A8B7C5E8-09E9-5B03-BEA1-61A40A7CAAAF

##### Type species.

*Cloresmus signoreti* Stål, 1860.

##### Revised diagnosis.

***Size and colour***: Small to medium-sized; dorsum densely punctate; coloration commonly ochraceous, brown, or olivaceous, often with a metallic greenish or brassy lustre.

***Head***: Somewhat flattened anteriorly; antenniferous tubercles not prominent; eyes prominent; ocelli red or pale; antennal segment I shorter than segment II, II longer than III, and segment IV typically the longest; rostrum short, not extending beyond the mesosternum; rostral segment I not longer than segment II, not exceeding the posterior margin of the head.

***Thorax***: Pronotum with a distinct collar, posterior angles rounded; lateral margins with verrucous protuberances; scutellum approximately equilaterally triangular; hemelytra with membrane sparsely veined; median fissure and M vein of the corium completely fused; hind coxae widely separated; males with a short spine on the outer edge of the hind coxae; hind femora thickened and ventrally spined, with males bearing a prominent long spine beyond the middle; hind tibiae prismatic.

***Abdomen***: Connexiva exposed dorsally, commonly with darker spots at the incisures; in the studied species, the male ultimate ventral sternite with a distinct medial incision; female sternite VII with a deep medial incision and fissura.

##### Distribution and diversity.

India (North Khasia Hills), Myanmar, Sumatra, China (Yunnan), Bangladesh, and Vietnam.

### Key to Species of *Cloresmus*

**Table:** 

1	Body dorsum with distinct dark metallic lustre	**2**
–	Body dorsum dull, yellowish-brown, brown, or reddish brown, without distinct metallic lustre; or with only pale shining	**7**
2	Eyes extremely large, length more than half of head length, and eyes contiguous to anterior border of pronotum	***C. boops* Blöte, 1936**
–	Eyes normal, not occupying half of head length	**3**
3	Antennal segments I–III with distinct olivaceous apices; segment IV ochraceous with a dark annulation at base and apex	***C. khasianus* Distant, 1901**
–	Antennal segments I–III without olivaceous apices; antennal colour pattern different	**4**
4	Antennal segment I piceous, segments II–III fuscous-brown, only segment IV pale ochraceous; antennae clothed with long hairs	***C. antennatus* Distant, 1908**
–	Antennae generally pale; or concolorous	**5**
5	Connexivum usually with black borders or dark olivaceous spots	**6**
–	Connexivum entirely yellow, without black spots	***C. jacobsoni* Blöte, 1936**
6	Antennae entirely luteous; connexivum luteous, spotted with dark olivaceous	***C. nepalensis* Westwood, 1842**
–	Antennae entirely ochraceous; connexivum yellow, with black borders at the ends of the segments	***C. javanicus* Westwood, 1842**
7	Pronotal anterior angles produced anteriorly and angulate; mid-ventral surface of the hind tibia with leaf-like broad spines	***C. tibiaspinus* sp. nov**.
–	Pronotal anterior angles flat, not produced; hind tibiae unarmed ventrally	**8**
8	Antennal segment IV concolorous	***C. similis* Dallas, 1852**
–	Antennal segment IV pale on its basal half	**9**
9	Body larger (13.6–18 mm); abdomen dorsum often purplish or red, with three distinct pale spots; connexivum with black or castaneous spots at incisures	***C. modestus* Distant, 1901**
–	Body smaller (c. 12 mm); colour dull yellowish-tan; head with an intraocular dark spot; hind femora brownish toward apex; hemelytra with a brassy sheen	***C. signoreti* Stål, 1860**

### Description of new species

#### Cloresmus tibiaspinus

Taxon classificationAnimaliaHemipteraCoreidae

Jiang & Bu sp. nov.

213A0106-DDFA-5246-BAF0-1574A3EA7422

https://zoobank.org/D49E98CA-62B2-426B-80D2-5245AF8CAFC0

Figs 4, 5

##### Chinese common name.

胫刺竹缘蝽.

##### Type material.

***Holotype***: • ♂, Liyue, Jingdong, Yunnan, 24.5093°N, 100.7595°E, 2001-XI-16, 1700 m, collector: Weibing Zhu. ***Paratypes***: • 3♂♂5♀♀, Liyue, Jingdong, Yunnan, 24.5093°N, 100.7595°E, 2001-XI-16, 1700 m, collector: Weibing Zhu; • 1♂1♀, Fangjiajing, Ailaoshan, Jingdong, Yunnan, 1984-V-14, collector: Leyi Zheng, Guoqing Liu.

##### Etymology.

The specific name, *tibiaspinus*, pertains to numerous members of this species that possess a spine on the tibia.

##### Diagnosis.

*Cloresmus tibiaspinus* sp. nov. can be diagnosed from all other species by the following morphological characters: (1) body size small (12.4–14.4 mm); (2) body yellowish-brown; (3) pronotal anterior angles produced anteriorly and angulate; (4) mid-ventral surface of the hind tibia slightly bent outwards, with or without one or two juxtaposed, large, leaf-like broad spines; and (5) posteroventral margin of the male genital capsule always trilobate with the mesial projection yellow and stout, and the lateral lobes rounded and brown.

##### Description.

**Figure 4.**
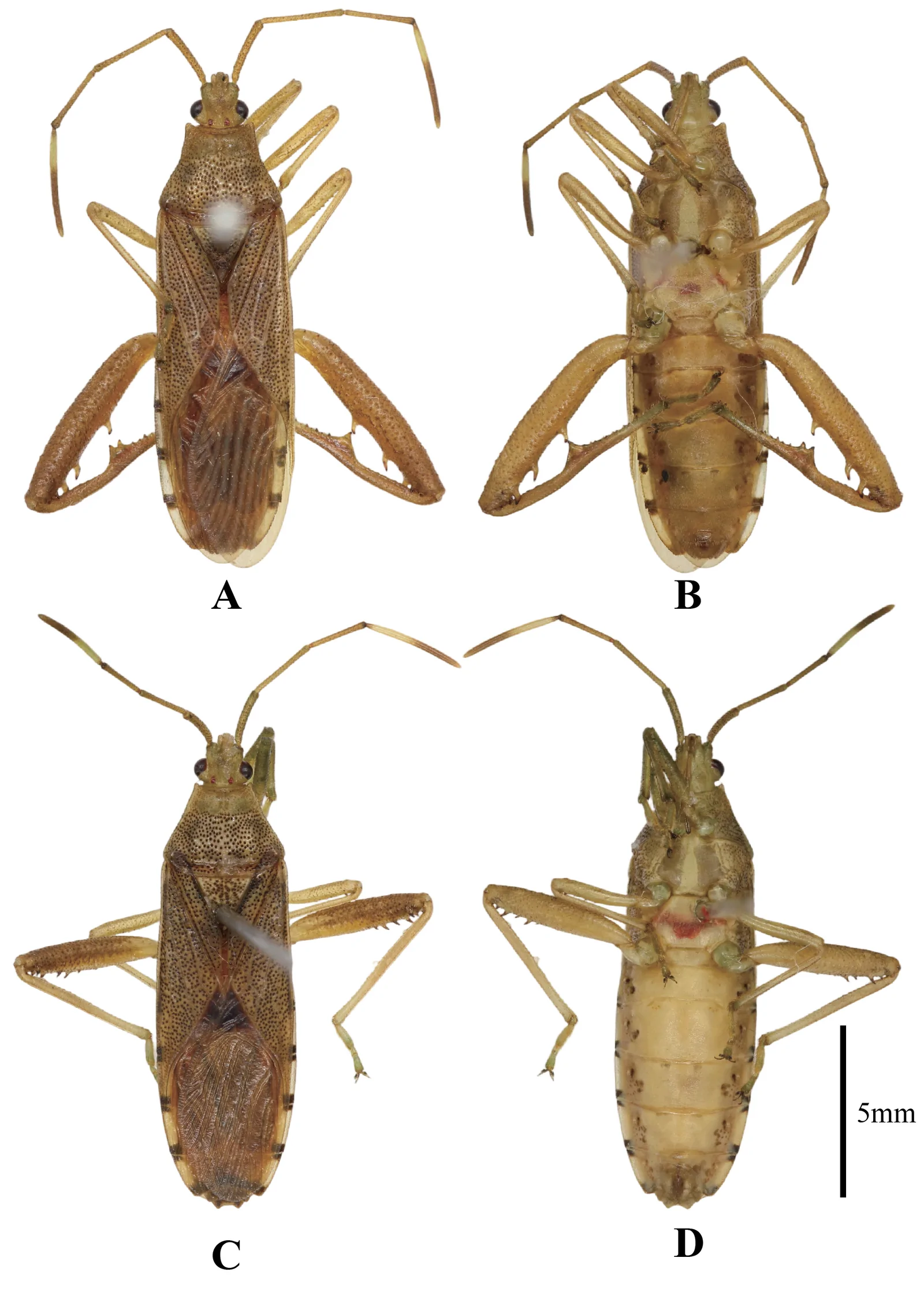
*Cloresmus tibiaspinus* sp. nov. **A**. Dorsal side of male; **B**. Ventral side of male; **C**. Dorsal side of female; **D**. Ventral side of female.

***Size and colour***: small in size, yellowish-brown, densely covered black punctures and short hairs (Fig. 4).

***Head***: Light yellowish-brown, without punctures, ventral surface lighter; vertex roughly level with eyes; tylus exceeding juga, with apices cleft; eyes brownish-black and prominent; ocelli red and slightly protruding; antenniferous tubercles prominent; antennal segments I and III approximately equal in length, segment IV the longest with basal half-light yellow and apical half greyish-black; buccula situated anterior to the antenniferous tubercles and produced anteriorly; rostrum covered with short hairs, reaching the anterior margin of the mesosternum, segment I not reaching the anterior margin of the eyes, midpoint of segment III approximately level with the posterior margin of the head; segments II and IV approximately equal in length and the longest.

***Thorax***: Pronotum yellowish-brown, with dense black punctures, nearly flat; calli covered with sparse fine little punctures, with a shallow pit on the midline; anterior angles produced anteriorly, angulate; humeral angles rounded and slightly projecting upward; lateral margins with indistinct ridges and verrucous protuberances; posterior margin slightly inclined downward, with a smooth transverse ridge; thoracic pleura with dense black punctures, sternum without punctures; scutellum nearly equilaterally triangular, with dense black punctures, and apex light yellow; corium of forewings yellowish-brown, densely covered with black punctures and short hairs, median fissure and M vein completely fused; membrane of forewings nearly transparent; scent gland orifice and evaporative area the same colour as the thoracic sternum; lateral side of hind coxa with a small protrusion; hind femur slightly swollen, basally bent outwards, with four large and curved spines on the inner margin, apex of these spines black; basal half of the dorsal surface of male hind femora armed with a row of small spines, approximately 4–5 in number; mid-ventral surface of the hind tibia slightly bent outwards, with or without one or two juxtaposed, large, leaf-like broad spines; in females, dorsal and ventral surfaces of the hind femur lacking prominent spines, and ventral surface of the hind tibia without leaf-like spines.

**Figure 5.**
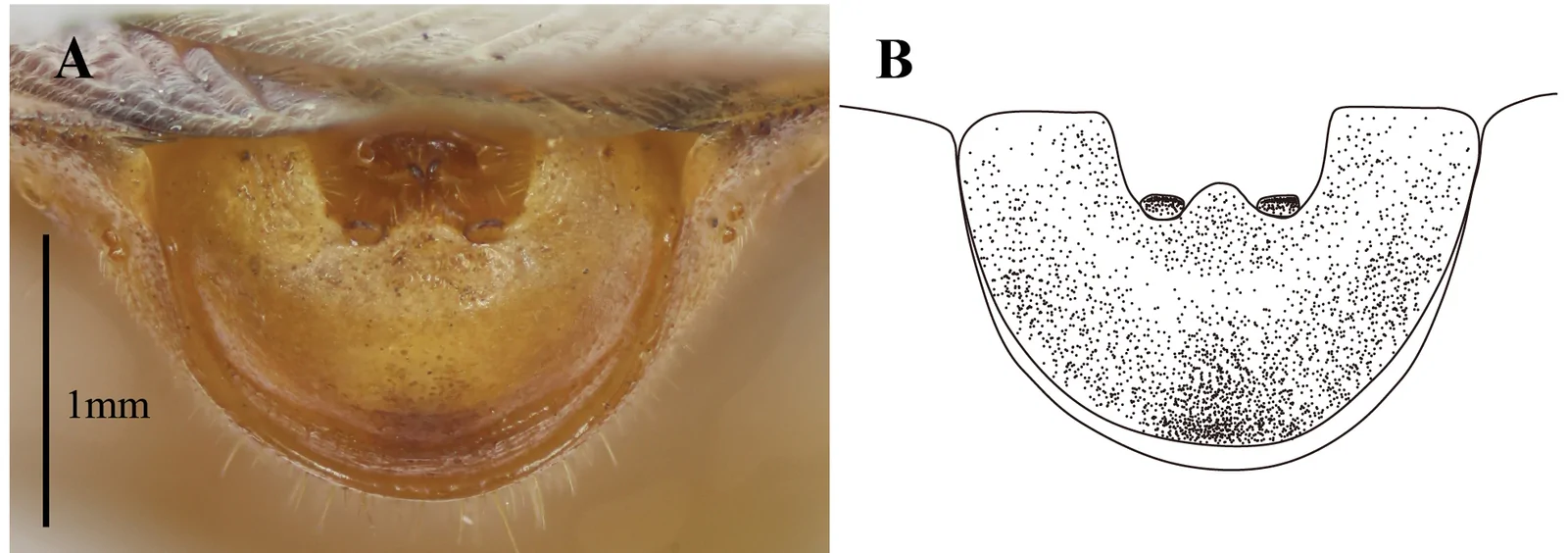
Male genital capsule of *Cloresmus tibiaspinus* sp. nov. **A**. Photograph of male genital capsule; **B**. Schematic diagram of male genital capsule.

***Abdomen***: Connexival segments light yellow medially, and black basally and apically; ventral abdomen and spiracles yellowish-brown; posteroventral margin of the male genital capsule with a deep U-shaped concavity, always trilobate with the mesial projection yellow and stout, and the lateral lobes rounded and brown (Fig. 5). Female abdominal sternite VII complete, both sides of the midline with plicae and a fissura; posterior part of sternite VII tightly joined.

##### Measurements

[in mm, ♂(*N* = 5)/♀(*N* = 6)]. Body length: 12.4–14.4; head length: 1.4–1.5, head width: 1.6–1.7; interocellar space: 0.3–0.4; antennal segments: I(1.3–2.0), II(1.8–2.4), III(1.5–2.0), IV(2.7–3.1); pronotal length: 2.2–2.8; pronotal width: 3.2–3.6; scutellar length: 1.4–1.7; scutellar width:1.5–1.8.

## Discussion

Our molecular phylogenetic study of the Oriental clade of the tribe Cloresmini reveals phylogenetic relationships that cannot be resolved relying solely on morphology. Within the genus *Notobitus*, *N. meleagris* and *N. elongatus* were previously considered to be closely related based on the length of antennal segment I, the straightness of the hind tibiae, and the coloration of the connexivum (Hsiao 1977). However, our research strongly supports that *N. montanus* and *N. elongatus* share a closer affinity to each other than to other species in this genus. The genus *Cloresmus* was previously considered a monophyletic group based on the shared character of possessing a tooth on the outer margin of the hind coxa (Hsiao 1977). However, a recently described new species in the genus *Notobitiella*, *N. bispina* (Jiang et al. 2022), demonstrated that this character is not exclusive to the genus *Cloresmus* (Jiang et al. 2022). The paraphyly of this genus remained consistent across both mitochondrial and nuclear datasets, strongly supporting the taxonomic validity of the newly erected genus *Gemenesis* gen. nov. Ecologically, both the new genus and *Notobitiella* are found at the apices of bamboo shoots. In contrast, while *Cloresmus* also feeds on bamboo leaves and shoots, its primary microhabitat is concealed within the basal sheaths of mature bamboo. This clear spatial and microhabitat divergence perfectly aligns with our molecular phylogenetic results (Jiang et al. 2024).

The exact phylogenetic relationships within the genus *Notobitus* require more nuclear gene data for verification. Furthermore, our study encompasses only 11 of the 33 recognized species in the Oriental clade of the tribe Cloresmini, which may limit our ability to fully resolve generic boundaries within the clade. Future work will focus on expanding taxon sampling from South and Southeast Asia (e.g., India, Bhutan, Myanmar, Malaysia, Indonesia, and Thailand). While morphological reviews of dried specimens from the Oceanian clade of Cloresmini have identified two genera and 11 species (Brailovsky 2006, 2007; Brailovsky and Barrera 2007), molecular data remain scarce. These specimens are often more than 100 years old, rendering their genomic DNA highly degraded. Consequently, obtaining fresh, alcohol-preserved samples is imperative for future studies to fully reveal the species diversity and evolutionary history of the tribe Cloresmini.

## Supplementary Material

XML Treatment for Cloresmini

XML Treatment for Gemenesis

XML Treatment for Cloresmus

XML Treatment for Cloresmus tibiaspinus

## Additional information

### Artificial Intelligence (AI) use

The authors accept full responsibility for the content of the manuscript, including the disclosure of any use of AI.

No AI tools were used in the preparation of this manuscript.

### Author ORCIDs

K. Jiang https://orcid.org/0000-0003-1932-7516

### Data availability

All of the data that support the findings of this study are available in the main text or Supplementary Information.

## Supplementary materials

Supplementary material 1 Support information Data type docx Explanation note **table S1**. Collectong information of sequenceing sample. **table S2**. Genes included in study, primer sequences and sources and locus-specific annealing temperatures (TA). **table S3**. Kimura 2-parameter (K2P) pairwise genetic distances based on complete COI gene. **fig. S1**. Phylogenetic tree of Cloresmini constructed by mitochondrial genome data (PCG12: all 13 PCGs with third codon positions removed). **fig. S2**. Phylogenetic tree of Cloresmini constructed by mitochondrial genome data (PCG123: all 13 PCGs). **fig. S3**. IQ tree of Cloresmini constructed by mitochondrial genome data (PCG123AA: the amino acid sequences of all 13 PCGs). **fig. S4**. Type species of the genus *Cloresmus*, *C. signoreti* Stål. https://binary.pensoft.net/file/1770088This dataset is made available under the Open Database License (http://opendatacommons.org/licenses/odbl/1.0/). The Open Database License (ODbL) is a license agreement intended to allow users to freely share, modify, and use this Dataset while maintaining this same freedom for others, provided that the original source and author(s) are credited.Author: Kun Jiang
