# Supplementary material for: Molecular and morphological evidence of the Oriental clade of the tribe Cloresmini (Hemiptera, Coreidae) reveals a new genus: Gemenesis gen. nov
Source: Zookeys. 2026 Sep 11;1291:223–38. doi: 10.3897/zookeys.1291.189894 (PMC13589295; doi:10.3897/zookeys.1291.189894)
Supplement: Supplementary file 1 [file zookeys-1291-223_article-189894__-s001.docx]

Table S1 Collectong information of sequenceing sample

| Species | Code | Location | Longitude | Latitude | Altitude | Date | Collector |
| --- | --- | --- | --- | --- | --- | --- | --- |
| *Anoplocnemis phasiana* | APH | Mengsong, Menghai, Yunnan province | 100.57 | 22.088 | 1451 | 2021-VII-10 | Juhong Chen |
| *Ochrochira pallipennis* | OPA | Menghun, Menghai, Yunnan province | 100.419 | 21.8693 | 1233 | 2021-VII-23 | Kun Jiang |
| *Physomerus grossipes* | PGR | Menglun, Menghai, Yunnan province | 101.225 | 21.952 | 588 | 2021-VII-13 | Kaibin Wang |
| *Cloresmus modestus* | CMOD1 | Nadana Village Salavan Laos | 106.384 | 15.7171 | 189 | 2019-VII-19 | Jiayue Zhou |
| *Cloresmus modestus* | CMOD2 | Menga, Menghai, Yunnan province | 100.3656 | 22.0955 | 1073 | 2021-VII-22 | Juhong Chen |
| *Cloresmus modestus* | CMOD3 | Ninger, Puer, Yunnan province | 101.0067 | 23.158 | 1168 | 2021-VII-21 | Juhong Chen |
| *Cloresmus sp1* | CMOD4 | Menghai, Menghai, Yunnan province | 100.4869 | 22.0208 | 1165 | 2021-VII-24 | Kun Jiang |
| *Cloresmus sp3* | CMOD5 | Menglun, Menghai, Yunnan province | 101.245 | 21.933 | 513 | 2021-VII-10 | Kun Jiang |
| *Cloresmus pulchellus* | CPU2 | Ninger, Ninger, Yuannan province | 101.1069 | 23.1062 | 1393 | 2021-VII-20 | Juhong Chen |
| *Cloresmus pulchellus* | CPU1 | Menghai, Menghai, Yunnan province | 100.3896 | 22.0456 | 1193 | 2021-VII-23 | Juhong Chen |
| *Cloresmus sp2* | CSP1 | Liyue, Jingdong, Yunnan province | 100.7595 | 24.5093 | 1700 | 2001-IX-16 | Weibing Zhu |
| *Cloresmus sp2* | CSP2 | Liyue, Jingdong, Yunnan province | 100.7595 | 24.5093 | 1700 | 2001-IX-17 | Weibing Zhu |
| *Cloresmus yunnanensis* | CYUN2 | Menga, Menghai, Yunnan province | 100.3611 | 22.1202 | 1068 | 2021-VII-22 | Kun Jiang |
| *Cloresmus yunnanensis* | CYUN1 | Menghun, Menghai, Yunnan province | 100.419 | 21.8693 | 850 | 2021-VII-23 | Kun Jiang |
| *Notobitiella bispina* | NBIS3 | Menga, Menghai, Yunnan province | 100.3636 | 22.1098 | 1071 | 2021-VII-22 | Kun Jiang |
| *Notobitiella bispina* | NBIS1 | Menghai, Menghai, Yunnan province | 100.3896 | 22.0456 | 1193 | 2021-VII-23 | Juhong Chen |
| *Notobitiella bispina* | NBIS2 | Menghai, Menghai, Yunnan province | 100.3896 | 22.0456 | 1193 | 2021-VII-23 | Juhong Chen |
| *Notobitiella elegans* | NELE1 | Menghai, Menghai, Yunnan province | 100.3636 | 22.1098 | 1071 | 2021-VII-22 | Juhong Chen |
| *Notobitiella elegans* | NELE2 | Menghai, Menghai, Yunnan province | 100.3896 | 22.0456 | 1193 | 2021-VII-23 | Juhong Chen |
| *Notobitus elongatus* | NELE1 | Mengban, Mengla, Yunnan province | 101.65 | 21.728 | 750 | 2014-VIII-20 | Penglei Guo |
| *Notobitus elongitus* | NELE2 | Menglun, Menghai, Yunnan province | 101.23 | 21.93 | 536 | 2021-VII-5 | Kun Jiang |
| *Notobitus elongitus* | NELE3 | Menglun, Menghai, Yunnan province | 101.267 | 21.914 | 533 | 2021-VII-10 | Kun Jiang |
| *Notobitus excellens* | NEXC2 | Puer, Yunnan province | 99.3534 | 22.2387 | 981 | 2016-VII-20 | Runxi Wang |
| *Notobitus excellens* | NEXC3 | Daluo, Menghai, Yunnan province | 100.0757 | 21.702 | 629 | 2013-VIII-11 | Lizhi Huo |
| *Notobitus excellens* | NEXC1 | Manwei, Menghai, Yunnan province | 100.3757 | 21.8089 | 1182 | 2021-VII-8 | Jiayue Zhou |
| *Notobitus meleagris* | NMEL3 | Nadana Village Salavan Laos | 106.384 | 15.7171 | 189 | 2019-VIII-19 | Jiayue Zhou |
| *Notobitus meleagris* | NMEL6 | Mengyang, Menghai, Yunnan province | 100.8875 | 22.157 | 798 | 2021-VII-27 | Kun Jiang |
| *Notobitus meleagris* | NMEL7 | Mengyang, Menghai, Yunnan province | 100.8895 | 22.157 | 798 | 2021-VII-27 | Juhong Chen |
| *Notobitus montanus* | NMON1 | Fenghuang, Zuoshui, Shaanxi province | 109.4005 | 33.5686 | 850 | 2016-VII-25 | Jiaqing Zhang |
| *Notobitus montanus* | NMON2 | Tianmushan, Zhejiang province | 119.4552 | 30.3289 | 650 | 2015-VII-26 | Wenbo Yi |
| *Notobitus sexguttatus* | NSEX1 | Luoshuai, Baisha, Hainan province | 109.546 | 19.0995 | 324 | 2017-VII-27 | Juanjuan Yuan |
| *Notobitus sexguttatus* | NSEX2 | Hin Hoeup Ventiane province Laos | 102.3348 | 18.6622 | 193 | 2019-VIII-24 | Yanfei Li |
| *Notobitus sexguttatus* | NSEX3 | Hin Hoeup Ventiane province Laos | 102.3348 | 18.6622 | 193 | 2019-VIII-24 | Yanfei Li |

Table S2. Genes included in study, primer sequences and sources and locus-specific annealing temperatures (TA).

| Gene | Primer name and sequence(5'-3') | Primer saurce | TA |
| --- | --- | --- | --- |
| 18S-1 rDNA | P1:CTGGTTGATCCTGCCAGTAGT | Campell et al., 1995 | 48-50℃ |
|  | P3:GGTTAGAACTAGGGCGGTATCT | Campell et al., 1995 |  |
| 18S-2 rDNA | P2:AGATACCGCCCTAGTTCTAACC | Campell et al., 1995 | 45-50℃ |
|  | P4:GATCCTTCTGCAGGTTCACC | Campell et al., 1995 |  |
| 28S rDNA | DD: GGGACCCGTCTTGAAACAC | Hillis and Dixon，1991 | 48℃ |
|  | FF: TTACACACTCCTTAGCGGAT | Hillis and Dixon，1991 |  |

Note: The complete 18S rDNA was amplified and sequenced in two contiguous regions using primer pairs 18S P1/18S P3 and 18S P2/18S P4.

Table S3. Kimura 2-parameter (K2P) pairwise genetic distances based on complete COI gene.

|  | 1 | 2 | 3 | 4 | 5 | 6 | 7 | 8 | 9 | 10 | 11 | 12 | 13 | 14 | 15 | 16 | 17 | 18 | 19 | 20 |
| --- | --- | --- | --- | --- | --- | --- | --- | --- | --- | --- | --- | --- | --- | --- | --- | --- | --- | --- | --- | --- |
| 1 CPUL1 |  |  |  |  |  |  |  |  |  |  |  |  |  |  |  |  |  |  |  |  |
| 2 CYUN1 | 0.123 |  |  |  |  |  |  |  |  |  |  |  |  |  |  |  |  |  |  |  |
| 3 NBIS1 | 0.150 | 0.145 |  |  |  |  |  |  |  |  |  |  |  |  |  |  |  |  |  |  |
| 4 NELE1 | 0.124 | 0.135 | 0.126 |  |  |  |  |  |  |  |  |  |  |  |  |  |  |  |  |  |
| 5 NEXC2 | 0.140 | 0.153 | 0.166 | 0.137 |  |  |  |  |  |  |  |  |  |  |  |  |  |  |  |  |
| 6 NEXC3 | 0.140 | 0.153 | 0.166 | 0.137 | 0.000 |  |  |  |  |  |  |  |  |  |  |  |  |  |  |  |
| 7 NEXC1 | 0.138 | 0.155 | 0.169 | 0.138 | 0.012 | 0.012 |  |  |  |  |  |  |  |  |  |  |  |  |  |  |
| 8 NSEX1 | 0.135 | 0.151 | 0.153 | 0.136 | 0.117 | 0.117 | 0.119 |  |  |  |  |  |  |  |  |  |  |  |  |  |
| 9 NMEL3 | 0.131 | 0.158 | 0.153 | 0.140 | 0.125 | 0.125 | 0.125 | 0.123 |  |  |  |  |  |  |  |  |  |  |  |  |
| 10 NELO1 | 0.144 | 0.160 | 0.162 | 0.139 | 0.129 | 0.129 | 0.129 | 0.124 | 0.120 |  |  |  |  |  |  |  |  |  |  |  |
| 11 NELO2 | 0.144 | 0.162 | 0.162 | 0.141 | 0.129 | 0.129 | 0.129 | 0.126 | 0.120 | 0.001 |  |  |  |  |  |  |  |  |  |  |
| 12 NELO3 | 0.143 | 0.161 | 0.162 | 0.140 | 0.128 | 0.128 | 0.128 | 0.125 | 0.119 | 0.001 | 0.001 |  |  |  |  |  |  |  |  |  |
| 13 NMON1 | 0.132 | 0.140 | 0.154 | 0.123 | 0.122 | 0.122 | 0.123 | 0.109 | 0.124 | 0.082 | 0.082 | 0.082 |  |  |  |  |  |  |  |  |
| 14 CSMA | 0.134 | 0.146 | 0.160 | 0.133 | 0.146 | 0.146 | 0.147 | 0.145 | 0.137 | 0.150 | 0.150 | 0.149 | 0.133 |  |  |  |  |  |  |  |
| 15 CMOD1 | 0.134 | 0.144 | 0.161 | 0.133 | 0.145 | 0.145 | 0.145 | 0.143 | 0.138 | 0.148 | 0.148 | 0.147 | 0.134 | 0.003 |  |  |  |  |  |  |
| 16 CMOD2 | 0.134 | 0.146 | 0.160 | 0.133 | 0.146 | 0.146 | 0.147 | 0.145 | 0.137 | 0.150 | 0.150 | 0.149 | 0.133 | 0.000 | 0.003 |  |  |  |  |  |
| 17 CMOD3 | 0.139 | 0.151 | 0.164 | 0.138 | 0.147 | 0.147 | 0.145 | 0.149 | 0.139 | 0.156 | 0.156 | 0.155 | 0.140 | 0.009 | 0.009 | 0.009 |  |  |  |  |
| 18 CBIN | 0.138 | 0.145 | 0.160 | 0.138 | 0.146 | 0.146 | 0.151 | 0.144 | 0.142 | 0.150 | 0.150 | 0.149 | 0.141 | 0.057 | 0.058 | 0.057 | 0.063 |  |  |  |
| **19 CNEW1** | **0.140** | **0.135** | **0.152** | **0.122** | **0.147** | **0.147** | **0.144** | **0.136** | **0.140** | **0.143** | **0.145** | **0.144** | **0.139** | **0.146** | **0.145** | **0.146** | **0.149** | **0.144** |  |  |
| **20 CNEW2** | **0.139** | **0.134** | **0.151** | **0.121** | **0.146** | **0.146** | **0.144** | **0.137** | **0.139** | **0.143** | **0.144** | **0.143** | **0.138** | **0.146** | **0.145** | **0.146** | **0.149** | **0.144** | 0.001 |  |

Figure S1. Phylogenetic tree of Cloresmini constructed by mitochondrial genome data (PCG12: all 13 PCGs with third codon positions removed). Numbers on the nodes are bootstrap values for IQ‐TREE and Bayesian posterior values for Mrbayes.

Figure S2. Phylogenetic tree of Cloresmini constructed by mitochondrial genome data (PCG123: all 13 PCGs). Numbers on the nodes are bootstrap values for IQ‐TREE. (Phylogenetic tree constructed by Mrbayes was same structure with Figure 1)

Figure S3. IQ tree of Cloresmini constructed by mitochondrial genome data (PCG123AA: the amino acid sequences of all 13 PCGs). Numbers on the nodes are bootstrap values for IQ‐TREE and Bayesian posterior values for Mrbayes.

Figure S4. Type species of the genus *Cloresmus, C. signoreti* Stål. Photographed by Birgit Jaenicke

Reference

Campbell, Bruce C., Jody D. Steffen‐Campbell, John T. Sorensen, and Raymond J. Gill. 1995. “Paraphyly of Homoptera and Auchenorrhyncha Inferred from 18S rDNA Nucleotide Sequences.” Systematic Entomology 20(3):175–94. doi: 10.1111/j.1365-3113.1995.tb00090.x.

Hillis, David M., and Michael T. Dixon. 1991. “Ribosomal DNA: Molecular Evolution and Phylogenetic Inference.” The Quarterly Review of Biology 66(4):411–53. doi: 10.1086/417338.
